# Methanol-to-hydrocarbons conversion over MoO_3_/H-ZSM-5 catalysts prepared *via* lower temperature calcination: a route to tailor the distribution and evolution of promoter Mo species, and their corresponding catalytic properties†
†Electronic supplementary information (ESI) available: more TEM images of post-run samples, CS Chem3D Model of zeolite and external surface MoO_3_, images and file (.c3xml). See DOI: 10.1039/c5sc01825k



**DOI:** 10.1039/c5sc01825k

**Published:** 2015-06-11

**Authors:** Bonan Liu, Liam France, Chen Wu, Zheng Jiang, Vladimir L. Kuznetsov, Hamid A. Al-Megren, Mohammed Al-Kinany, Saud A. Aldrees, Tiancun Xiao, Peter P. Edwards

**Affiliations:** a KACST – Oxford Petrochemical Research Centre (KOPRC) , Inorganic Chemistry Laboratory , Department of Chemistry , University of Oxford , Oxford , UK . Email: Peter.edwards@chem.ox.ac.uk ; Email: xiao.tiancun@chem.ox.ac.uk; b Petrochemical Research Institute , King Abdulaziz City for Science and Technology , P. O. Box 6086 , Riyadh 11442 , Kingdom of Saudi Arabia; c Department of Materials , University of Oxford , Oxford , UK

## Abstract

A series of MoO_3_/H-ZSM-5 (Si/Al = 25) catalysts were prepared *via* calcination at a lower-than-usual temperature (400 °C) and subsequently evaluated in the methanol-to-hydrocarbon reaction at that same temperature. The catalytic properties of those catalysts were compared with the sample prepared at the more conventional, higher temperature of 500 °C. For the lower temperature preparations, molybdenum oxide was preferentially dispersed over the zeolite external surface, while only the higher loading level of MoO_3_ (7.5 wt% or higher) led to observable inner migration of the Mo species into the zeolite channels, with concomitant partial loss of the zeolite Brønsted acidity. On the MoO_3_ modified samples, the early-period gas yield, especially for valuable propylene and C_4_ products, was noticeably accelerated, and is gradually converted into an enhanced liquid aromatic formation. The 7.5 wt% MoO_3_/H-ZSM-5 sample prepared at 400 °C thereby achieved a balance between the zeolite surface dispersion of Mo species, their inner channel migration and the corresponding effect on the intrinsic Brønsted acidity of the acidic zeolite. That loading level also possessed the highest product selectivity (after 5 h reaction) to benzene, toluene and xylenes, as well as higher early-time valuable gas product yields in time-on-stream experiments. However, MoO_3_ loading levels of 7.5 wt% and above also resulted in earlier catalyst deactivation by enhanced coke accumulation at, or near, the zeolite channel openings. Our research illustrates that the careful adoption of moderate/lower temperature dispersion processes for zeolite catalyst modification gives considerable potential for tailoring and optimizing the system's catalytic performance.

## Abstract 2

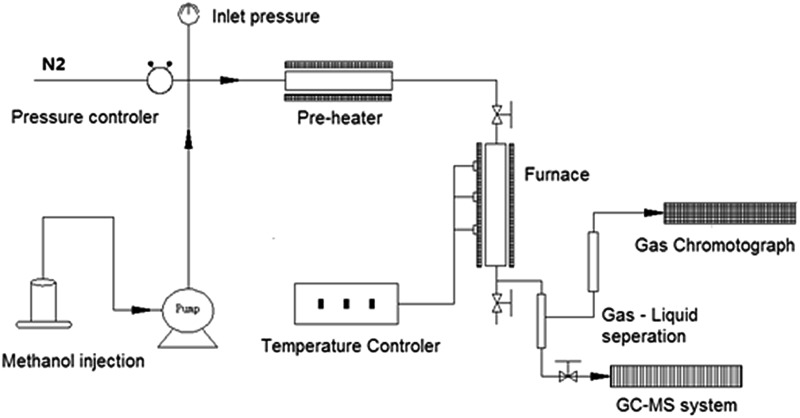
A series of MoO_3_/H-ZSM-5 catalysts were prepared *via* calcination at a lower-than-usual temperature and evaluated.

## Introduction

The pivotal role of methanol in any future sustainable energy scenario has been extensively discussed under the “Methanol Economy” concept.^1^ This science also figures strongly in visions of the large scale production of carbon-neutral synthetic fuels for the decarbonisation of transportation as well as in the synthesis of fuels and important organic chemicals derived from air, water and sustainable energy. Methanol therefore serves both as a highly effective energy carrier, as well as an abundant, ubiquitous resource and a “bridge chemical” for the synthesis of important liquid fuels and hydrocarbon products. The Methanol-to-Gasoline (MTG) process technology, first developed by the Mobil Company, was advanced as an economic solution to transportation fuel in the 1970's oil crisis.^2^ In this methanol conversion process, olefins and aromatics are competitor products; accordingly, the reaction^3,4^ (more broadly defined as Methanol-to-Hydrocarbons, MTH) is divided into 3 sub routes including MTG^5^ (typically taken as gasoline-boiling-range hydrocarbons), Methanol-to-Olefins^6,7^ (MTO) and Methanol-to-Aromatics^8^ (MTA, typically focused on the production of important aromatics *e.g. para*-xylene). The solid acid catalyst ZSM-5 zeolite, H-ZSM-5 was previously found to exhibit excellent catalytic performance in the early stage researches on MTH reaction.^9–11^ Other types of zeolite have also been studied extensively; *e.g.*, H-SAPO-34 zeolite of the LTA structure (having smaller pore openings ideal for light olefin products) has been shown to be extremely suitable for the production of ethylene and propylene.^12,13^

The generally accepted mechanism of MTH reaction has been based on a so-called ‘Hydrocarbon Pool’ (HP) mechanistic route in which poly-methylated benzenes are considered to be the major active intermediates (part of the catalysis centre, promoted the reaction by series of alkylation and de-alkylation steps on the benzene ring) in the reaction.^14,15^ However, the reaction mechanism appears rather different for ZSM-5 as compared to that for other zeolites (*e.g.* H-SAPO-34), as a methylation process of primary olefin products and subsequent intra conversion (*e.g.* cracking) between previously-formed olefins have also been involved as a parallel route to the HP mechanism (contributing to only the C_3_^+^ olefins, ethylene formation is mechanistically not involved in this route).^16^ Importantly, we note that this mechanism could be a tool to adjust the final MTH performance and product distribution by precisely ‘tailoring’ the location and nature of the catalytically active sites, their dispersion on, and in, the supporting zeolite structure as well as permitting experimental control of the resulting product diffusion.

Molybdenum oxide species have been widely used as catalyst promoters in methane activation, with the MoO_3_/H-ZSM-5 catalyst considered to be the most effective for methane aromatization.^17–20^ One reason that has been advanced is the important activation of C–H bonding in the CH_4_ molecule by the resulting molybdenum carbides or oxy-carbide catalysts (derived originally from MoO_3_).^21^ The actual spatial location and distribution of any MoO_3_ or other Mo-based species in H-ZSM-5 relies precisely on the conditions used in the catalyst preparation, especially the process temperature and the degree of coverage of MoO_3_ on the zeolite surface.^22^ Moreover, a relatively low calcination temperature (*e.g.* 300 °C) is sufficient for the decomposition of ammonium hepta molybdate (the precursor, denoted as AHM) into MoO_3_ clusters (or oligomers), leading to formation and various dispersions of MoO_3_ molecular entities or nano-to-micro scale molybdenum oxide particles on the zeolite outer surface.^22–24^ Temperatures in the range of 350–400 °C, are known to initiate the processes of MoO_3_ species sublimation and also the beginnings of MoO_3_ diffusion into the host zeolite support.^25^ Borry *et al.*, noted that temperatures above 500 °C are required for MoO_3_ species (in terms of various molecular clusters and oligomers) to completely migrate into the ZSM-5 zeolite channels *via* surface transportation in gas phase.^22^ At these temperatures, and as a result of the MoO_3_ inner migration, (Mo_2_O_5_)^2+^ dimer structures were proposed to form within the zeolite channels, which would be subsequently reduced by CH_4_ to form molybdenum carbides (MoC_*x*_) as the active catalytic sites for the ensuing C–H bond activation and olefin aromatization. However, the formation of such a (Mo_2_O_5_)^2+^ or carbide species within the zeolite channel would then naturally reduce the intrinsic Brønsted acidity of the host (support) zeolite which is also required for the methanol conversion and later aromatization steps.^26^

Methanol conversion over zeolite catalysts usually employs a temperature range between 350–450 °C, which is lower than the normally required calcination temperature (often 550 °C) for impregnated catalysts. In this investigation, we have prepared a range of MoO_3_/H-ZSM-5 catalyst samples prepared *via* lower temperature calcinations, specifically, 400 °C, and compared to the often-used 550 °C, and examined the resulting catalyst performance at that same lower temperature. The research takes advantage of the fact from earlier studies,^20,22,27^ together with an examination of vapour pressure studies, that MoO_3_ would be primarily dispersed on the *exterior* zeolite surface at this reduced processing temperature, maintaining the majority of the inner channel zeolite Brønsted acidity at a desired level. Furthermore, only high MoO_3_ loading levels may lead to significant inner migration of Mo species to form the potential catalytically active sites which would promote methanol conversion and a potential higher yield of valuable hydrocarbon products. The potential benefit of such a lower temperature may result in reduced energy costs, and, importantly, more freedom on tailoring the distribution of Mo species in the zeolite system in the catalyst preparation process.

Here, we mainly focus on the desirable MTO products (ethylene, propylene and C4 mixtures), as well as the major aromatic products (benzene, toluene, and xylenes, denoted as BTX). At an appropriate MoO_3_ loading level, a balance between the distribution of the outer dispersed Mo species, and the inner Mo active sites, as well as the zeolite Brønsted acidity may be carefully tailored for a particular, optimized target product distribution. This advance will, we believe, assist towards targeted, more sustainable hydrocarbon production.

## Experimental

### Preparation of catalyst

Three MoO_3_/H-ZSM-5 (Si/Al = 25) samples of different MoO_3_ loading levels (5 wt%, 7.5 wt%, and 10 wt%) were prepared *via* incipient wetness impregnation using aqueous solution of ammonium hepta molybdate (Sigma, USP specifications), followed by dehydration at 100 °C in a drying cabinet for 24 h and subsequent calcination at 400 °C for 5 h in static air. For direct comparison, a 7.5 wt% MoO_3_ H-ZSM-5 sample was also prepared *via* 500 °C calcination. The ZSM-5 is supplied by WENFENG Co. Ltd (China), packed in NH_4_-ZSM-5.

### Characterization methods

The dispersion of Mo species and zeolite crystalline phase change were analysed with X-ray powder diffraction (XRD), Fourier-transform infrared spectroscopy (FT-IR) and laser Raman spectroscopy. Ammonium-temperature-programmed desorption (NH_3_-TPD) was employed to investigate the changes on zeolite acidity distribution. With the help of Brunauer–Emmett–Teller (BET) analysis and Scanning Electron Microscope (SEM), the surface properties and morphology of the prepared catalysts were also examined. Transmission Electron Microscope (TEM) measurements were applied in the research of zeolite surface Mo species and coke deposition observation. Coke formation was also evaluated with thermogravimetric analysis (TGA).

XRD data was obtained with a PANalytical X'Pert PRO Diffractometer using Cu Kα1 radiation, the scan diffraction angle from 5° to 90° (2*θ* angular range) and a scan rate of 0.8° min^–1^ in 2*θ*.

FT-IR data were collected using a Perkin-Elmer Spectrum RX1 FT-IR spectrometer. Before analysis, the pressed sample tablets (diluted with KBr) were dehydrated at 300 °C in air for 12 hours to remove the moisture adsorbed by the zeolite. The tablets were then rapidly transported onto the FT-IR sample holder at 150 °C for direct analysis in an attempt to minimize the disturbance by the adsorbed water molecules.

Laser Raman spectra were recorded with a Perkin-Elmer Raman Station 400F Raman Spectrometer. The powder sample was supported on a piece of clean glass for scanning.

The NH_3_-TPD analysis was performed using a TP-5078 Auto TPD system (Xianquan Co. Ltd, China). The loaded samples (500 mg) were carefully pre-treated in N_2_ at 300 °C for 2 h to remove the adsorbed moisture. The NH_3_ adsorption (NH_3_ : N_2_ = 1 : 3) performed at 120 °C for 30 min, and the system was then cooled down to room temperature for 1 h in pure N_2_. The samples were then heated at 8 °C min^–1^ from room temperature to 600 °C, the desorbed NH_3_ was recorded with a Thermal Conductivity Detector (TCD).

The BET analysis proceeded with a Micromeritics Gemini VI BET surface area analyzer. The total surface area was calculated from adsorption data at *p*/*p*_0_ = 0.05 and the total volume of all the tested samples was measured at *p*/*p*_0_ = 0.995.

The SEM images were obtained with a JEOL scanning microscope 840F (JSM840F). The sample powder was deposited onto the dust-free scanning platform and trapped on the surface before analysis. The resolution was carefully adjusted to make sure the sharp and clear edge of the zeolite crystalline grains could be observed with as many as optical details of the external surface. TEM measurements were undertaken using a JEM-2100UHR microscope (200 kV).

A SDT Q600 (TA instruments) thermogravimetric (TGA) analyzer was employed to determine the coke content. The coke amount is determined by weight loss during temperature programmed oxidization in air from 20 to 1120 °C (temp. ramp 10 °C min^–1^). 50 mg of spent sample was used each time.

### Catalyst testing

**Fig. 1:**
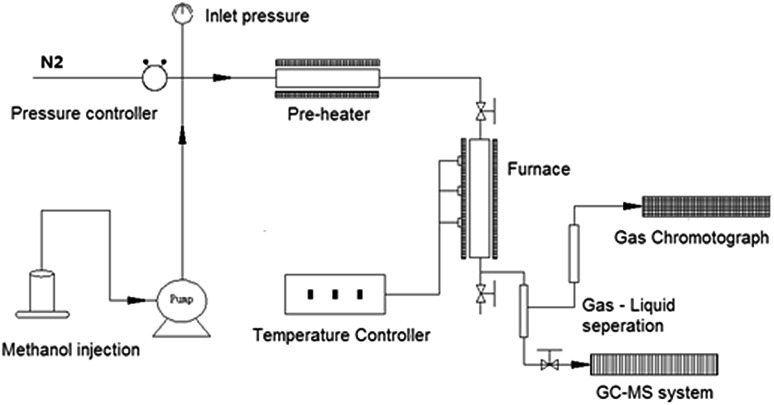
Schematic representation of the reactor, and testing & analysis system.

Catalyst performance tests were carried out in a fixed bed reactor system (Beijing KLYT Co. Ltd, China) as shown schematically in Fig. 1. Methanol was injected using a HPLC pump and vaporized at 150 °C before entering the reactor. Dry nitrogen (20 ml min^–1^) was used as carrier gas and internal standard for gas product quantification. The reaction took place in a tubular stainless steel reactor at 400 °C under the pressure of 1 atm; typically, 1.0 g of catalyst was tested each time when a methanol Weight Hourly Space Velocity (WHSV) of 2 h^–1^ was applied.

The gas products were directly transferred to a GC system (Shimadzu GC-2010SE) and analyzed with thermal conductivity detector (TCD) for non-hydrocarbons and flame ionized detector (FID) for hydrocarbons every 30 minutes, after methanol injection started. The gas product separation in GC system employed a RESTEK MXT-1HT column.

The liquid products were collected after the reaction, separated into water phase (water was generated in the reaction) and oil phase (hydrocarbon products) *via* still standing, both of which were then analyzed with an offline GC-MS system using FID detector (Shimadzu GCMS-QP2010 Ultra High-end). Separation of different liquid components proceeded in a SHIM-5MS column.

The methanol conversion was defined by amount of methanol consumed compared to the total methanol fed. The amount of methanol remaining in the liquid product (both oil phase and water phase) is analyzed with offline GC-MS. The gas product time-on-stream (TOS) yield (mol%) was calculated by comparing the instantaneous gas product methanol consumption (mol min^–1^) with methanol injection rate (mol min^–1^). The corresponding benzene, toluene, and xylenes (BTX) selectivity (taken after 5 hours) was calculated from the methanol consumption of selected products (mol) and total methanol converted (mol). The definitions shown below are based on previous researches and modified to the current research.^2,9,10,12,13,26^ 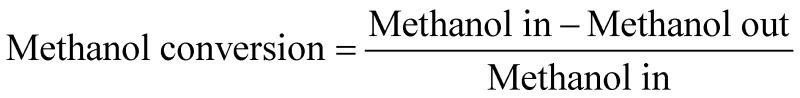 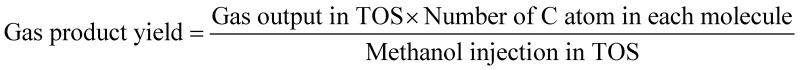 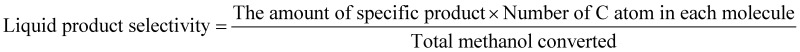


## Results and discussion

### X-ray diffraction (XRD) analysis of the as-prepared catalysts

**Fig. 2:**
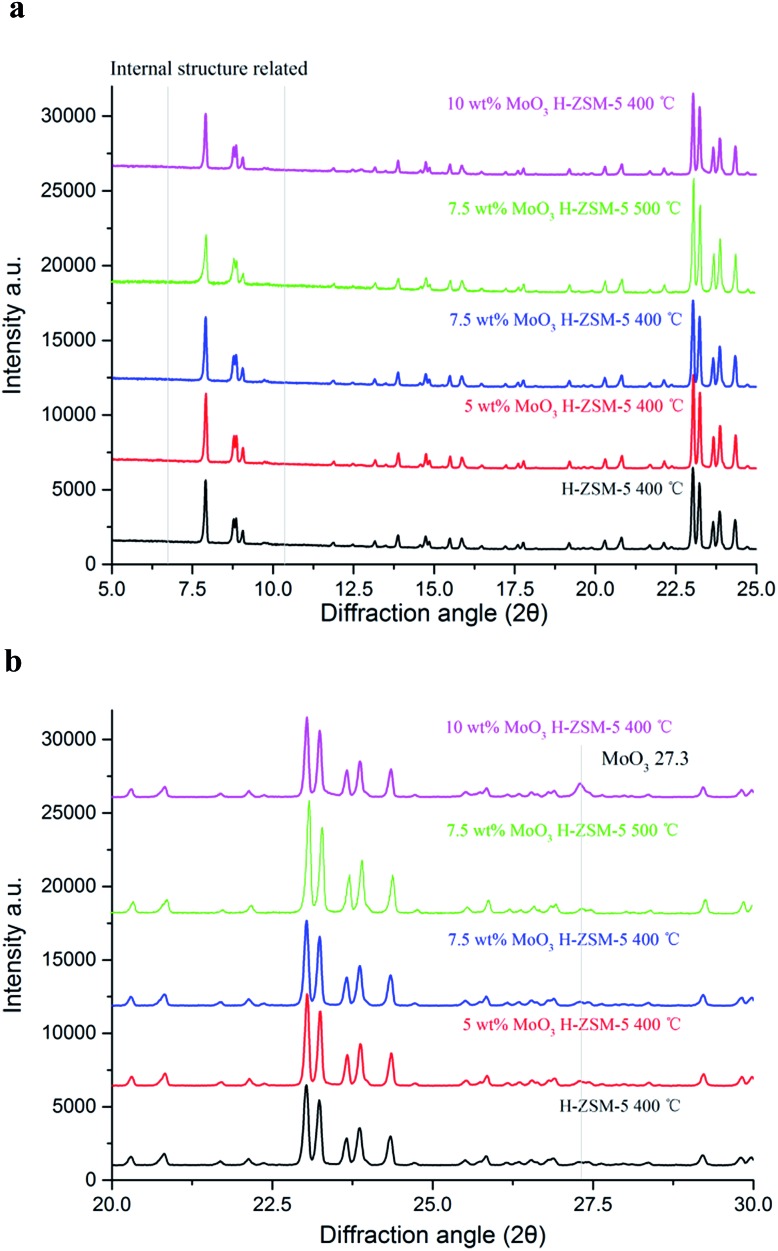
(a) X-ray diffraction patterns (2*θ* = 5–25°) of MoO_3_/ZSM-5 samples with various MoO_3_ loadings and prepared at 400 °C/500 °C. (b) X-ray diffraction patterns (2*θ* = 20–30°) of MoO_3_/ZSM-5 samples with various MoO_3_ loadings and prepared at 400 °C/500 °C.

XRD patterns of the as-prepared MoO_3_ loaded samples, as well as the parent H-ZSM-5 are shown in Fig. 2a and b. There are no obvious differences in the XRD patterns between MoO_3_/H-ZSM-5 and the original H-ZSM-5 samples, highlighting the critical issue of only minimal (if any) change of the supporting zeolite framework by MoO_3_ modification at the processing temperature of 400 °C (and 500 °C for 7.5 wt% MoO_3_). We note also that the two groups of XRD peaks located at 5° < 2*θ* < 10°, are known to be related to the intrinsic zeolite channel inner structure;^28^ and they are also well maintained on all the modified samples, showing limited disturbance of the zeolite channel inner framework by the impregnated Mo species under our preparative conditions.

In earlier work, it has been reported that 500 °C calcination and higher Mo concentrations (4 wt% or higher for MoO_3_) resulted in significant migration of the Mo–Oxy species into the zeolite channels, leading to potential dealumination of the zeolite inner framework and subsequent loss of zeolite Brønsted acidity. In support of this conjecture, crystalline Al_2_(MoO_4_)_3_ was detected both by ^27^Al NMR and XRD in various studies.^22,27^

In this work, characteristic XRD reflections arising from crystalline Al_2_(MoO_4_)_3_ were not observed. In contrast, in an expanded scan, a small peak at 2*θ* = 27.3°, assigned to crystalline MoO_3_, was observed for the 10 wt% MoO_3_/H-ZSM-5 sample (Fig. 2b). At lower loading levels, no peak attributed to bulk crystalline MoO_3_ was detected, and this points to the occurrence of possible inner migration, and an effective, but thin, dispersion of the crystalline molybdenum oxide species, probably as a monolayer of (MoO_3_)_*x*_ oligomers on the zeolite external surface. Supporting evidence for our assertion comes from other studies of the decomposition of AHM species into various MoO_3_ species spreading on the zeolite external surface at 300 °C or higher temperature, as reported by Borry and co-workers.^22^ A colour change from white to a homogeneous light-lime-green was also observed when the loading of Mo species increased, suggesting the dispersion of MoO_3_ species over the zeolite outer surface.

### Raman spectra

**Fig. 3:**
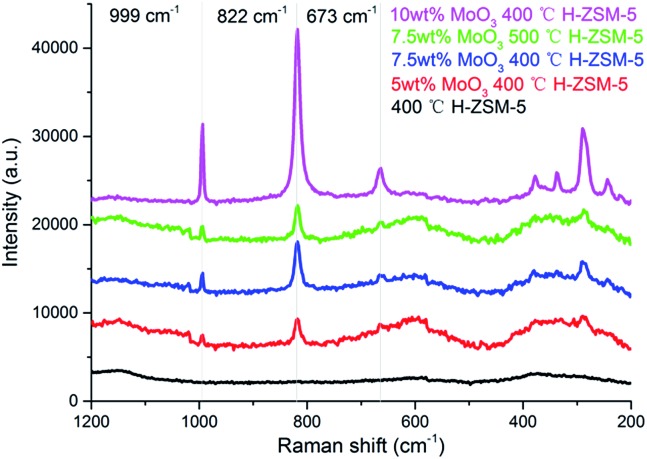
Laser Raman spectra of the as prepared MoO_3_/ZSM-5 samples and H-ZSM-5 and prepared at 400 °C/500 °C.

It has been reported that both the “bulk” MoO_3_ and various MoO_*x*_ phases (where the Mo/O stoichiometry varies) could coexist on the Al_2_O_3_ support but for zeolite aluminosilicate systems this is not yet confirmed.^29^ The laser Raman spectra of various prepared samples are shown in Fig. 3. The band at 955 cm^–1^, arising from MoO_*x*_ species is not observed. In contrast, three Raman resonance bands at 673 cm^–1^, 822 cm^–1^, and 999 cm^–1^ are identified for the MoO_3_ structure.^29^ These results show that there is predominantly dispersion of MoO_3_ occuring on the zeolite surface under the present experimental conditions. According to the XRD results, it is also suggested that these Raman observable MoO_3_ structures could be small clusters or oligomers of MoO_3_ at lower loading levels (7.5 wt% or lower), and the XRD observable “bulk” crystal phase of MoO_3_ would only emerge at significantly higher loading levels (10 wt% or above). The Raman band of surface MoO_3_ on 7.5 wt% MoO_3_ H-ZSM-5 (500 °C cal.) is relatively weaker than the 400 °C treated sample, suggesting more efficient inner migration of MoO_3_ into the zeolite interior.

### Fourier transform-infra red (FT-IR) spectra

**Fig. 4:**
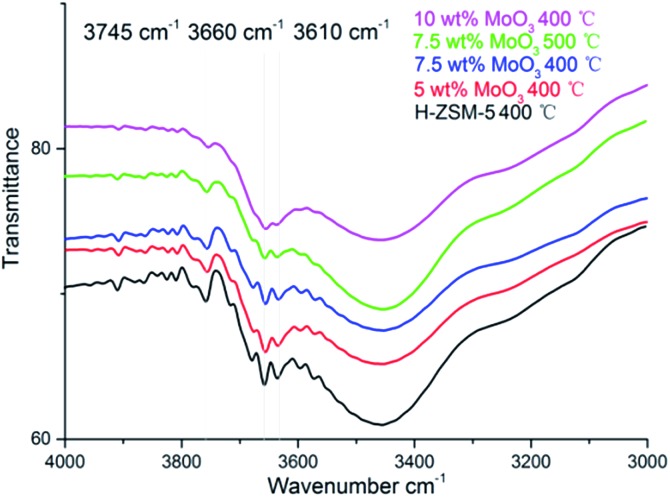
Fourier transform-IR spectra of hydroxyl group regions on the parent H-ZSM-5 and MoO_3_-loaded samples prepared at 400 °C/500 °C.

The FT-IR spectra of H-ZSM-5 and MoO_3_ loaded samples are given in Fig. 4. The band located at 3745 cm^–1^ is assigned to the –O–H vibration of the silanol groups (Si–OH) mostly on the external surface of the zeolite;^26^ they are considered to be a typical terminal structure of a zeolite crystal. The silanol groups, as part of the zeolite structure, are considered to have relatively weak acidity (Brønsted acidity) in contrast to the Al–OH moiety but sometimes they play a role as proton donor in catalytic reactions.^30^ The bands ranging from 3660 cm^–1^ to 3610 cm^–1^ in Fig. 4 are ascribed to the Al-bonded hydroxyl groups. The peak at 3660 cm^–1^ is related to the extra framework Al–OH groups (weak Brønsted acidity) while the peak at 3620 cm^–1^ is associated with the strong Brønsted acid sites those are of Si–OH–Al structure, which is an integral part of the zeolite framework.^26^ Comparing with other hydroxyl groups, these framework Brønsted acid sites are mostly located inside the zeolite channel.^31^ As it was previously shown, the migration of MoO_3_ species into zeolite channels leaded to a dramatic decrease in the number of these Brønsted acid sites.^17,22^

As shown in Fig. 4, at low MoO_3_ loading levels (typically 5 wt% and below) the zeolite hydroxyl groups were well maintained. It is assumed that at 400 °C the interaction between zeolite structure and the adsorbed MoO_3_ species is comparably moderate. This is also reflected by the well maintained zeolite framework, as shown in the XRD patterns. The loss of framework Brønsted acid sites, and other Al–OH groups, was observed when the MoO_3_ loading level increased to 7.5 wt%, whereas the loss of Si–OH groups was relatively moderate. When the loading level of MoO_3_ rose up to 10 wt%; an observed intensive decrease of all the hydroxyl groups was shown in the FT-IR spectra, almost certainly arising from the interference of MoO_3_ species at this high loading level. The 7.5 wt% MoO_3_/H-ZSM-5 sample lost slightly more acid sites at the 500 °C temperature, illustrating the promoting effect of the preparation temperature for the inner migration of Mo species. The system also has less silanols, possibly due to the promoted zeolite surface transportation and dispersion of MoO_3_ before the inner migration.

The loss of some degree of Brønsted acidity in MoO_3_/HZSM-5 catalysts at loading levels of 7.5 wt% and above suggests that some MoO_3_ migrates into the zeolite channels, even at 400 °C, and is probably enhanced in the high MoO_3_ concentration samples. On the other hand, the Si–OH (silanol) groups were not disturbed until the bulk MoO_3_ crystal emerged (on the 10 wt% MoO_3_/H-ZSM-5, Fig. 2b), showing a preference of the MoO_3_ species to bind with the strong acidic sites.

**Fig. 5:**
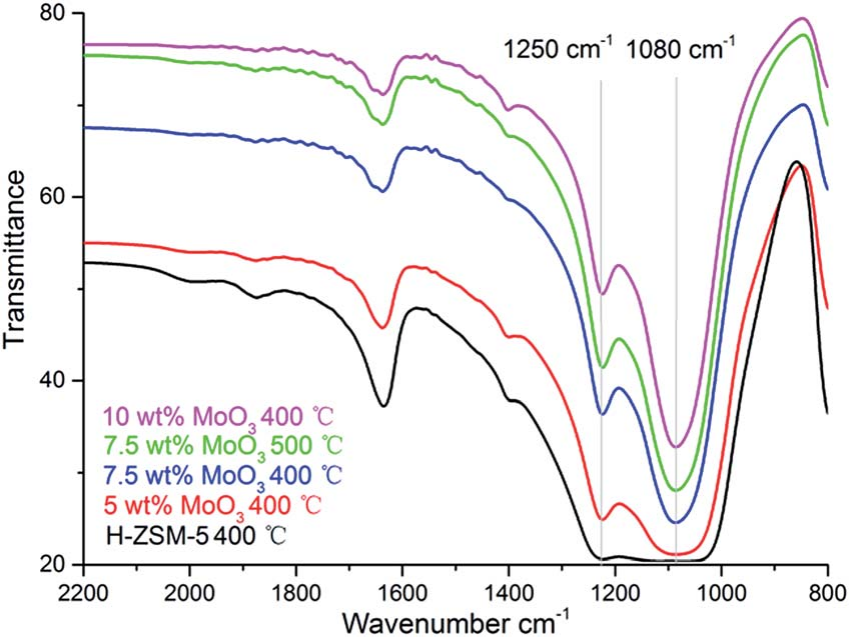
Fourier Transform-IR spectra of H-ZSM-5 samples with different MoO_3_ loading levels prepared at 400 °C/500 °C.

The formation of MoO_3_ phase on the prepared samples is also suggested by the FT-IR, as shown in Fig. 5. The bands at 1250 cm^–1^ and 1080 cm^–1^ are characteristic of the MoO_3_ vibrational models although the precise location of the band varies in different studies.^23^

### NH_3_-temperature programmed desorption (NH_3_-TPD)

**Fig. 6:**
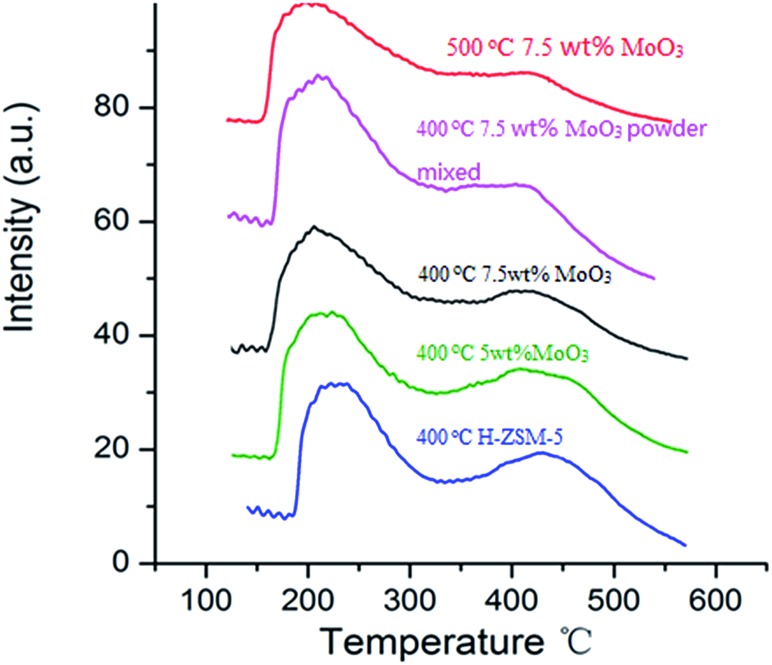
NH_3_-temperature programmed desorption profiles of MoO_3_/H-ZSM-5 samples prepared *via* different conditions (impregnation/powder mixing, 400–500 °C) together with the original H-ZSM-5 sample (400 °C).

NH_3_-TPD studies are routinely used to study the acidity of solid acid catalysts and their subsequent modification after particular thermal and chemical treatments.^17,32^ The NH_3_ desorption profile of original H-ZSM-5 (activated from NH_4_-ZSM-5 *via* 400 °C calcination, used as reference) exhibits 2 distinct peaks at different temperatures (Fig. 6). A desorption peak at around 220 °C is assigned to the NH_3_ molecules adsorbed on weak acid sites (Si–OH, extra framework Al–OH) while the peak at 450 °C is attributed to the NH_3_ molecules on the strong acidic Brønsted acid sites (mainly located at zeolite inner channels).^32,33^

Two 7.5 wt% MoO_3_/H-ZSM-5 samples were prepared *via* impregnation technique with AHM and calcination at 400 °C and 500 °C for 5 h, respectively. Another sample was prepared by mechanical mixing 7.5 wt% of MoO_3_ with 92.5 wt% of NH_4_-ZSM-5 (room temperature overnight stirring in water, dried at 100 °C) and subsequent calcination at 400 °C. The MoO_3_ used in this mechanical mixture was prepared by the decomposition of AHM at 400 °C. The selected 7.5 wt% doping level is based on an observed optimized catalysis discussed in the following sections. In order to compare the effects of different MoO_3_ concentrations on zeolite acidity distribution, a 5 wt% MoO_3_/H-ZSM-5 sample, synthesized *via* impregnation and calcination at 400 °C, was also tested.

Comparing with the original H-ZSM-5 catalyst, from the NH_3_-TPD profile of the 7.5 wt% MoO_3_/H-ZSM-5 sample (made *via* impregnation, and 400 °C calcined) we observed a partial decrease on the number of desorbed NH_3_ as reflected by the change of profile at around 450 °C, where observable erosion of peak was shown. This indirectly reflected the loss of strong acid sites by MoO_3_ loading. The 7.5 wt% MoO_3_/H-ZSM-5 sample prepared *via* physical mixing exhibited somewhat a similar result but lost more strong acid sites.

Importantly, there is no obvious difference between the original H-ZSM-5 catalyst and the 5 wt% MoO_3_/H-ZSM-5 sample calcined at 400 °C. More obvious is the loss of Brønsted acid sites observed for the impregnation prepared 7.5 wt% MoO_3_/H-ZSM-5 sample, calcined at 500 °C. For this, the NH_3_ desorption peak at the 450 °C temperature became relatively weaker compared with other samples. The NH_3_-TPD results of the 400 °C calcined samples are in accord with the above FT-IR analysis that at the 7.5 wt% loading level, part of the MoO_3_ based, or derived species appears to have migrated into the zeolite channels leading to some loss of zeolite Brønsted acidity. We note that the concentration of MoO_3_ species played an important role in affecting the Brønsted acidic site. The NH_3_-TPD results also show that more effective migration of MoO_3_ into ZSM-5 zeolite channel occurred at a higher temperature of 500 °C, which is in agreement with the previous studies of these catalyst materials.^17,22,32^

We also note that the sites of “weak acidity” were not noticeably disrupted by the MoO_3_ species since the desorption peak at around 220 °C was well maintained for each sample. This is also confirmed by the presence of Si–OH groups in the FT-IR studies. Thus, it can be concluded that the interaction between the adsorbed MoO_3_ species and these weak acidic hydroxyls mostly on the external ZSM-5 surface is comparatively weak. The exact nature of Mo species on the zeolite external surface prepared *via* 400 °C calcination needs further examination, but at present we assign this as a monolayer dispersion of (MoO_3_)_*n*_ clusters and oligomers.

Thus, both the calcination temperature and the MoO_3_ concentration have a considerable impact on the scale and extent of the MoO_3_ coverage on the exterior surface of the H-ZSM-5 zeolite and also on its migration into the interior channel of the zeolite support/host and the attendant acid properties of the resulting catalyst.

### Scanning electron microscopy (SEM)

**Fig. 7:**
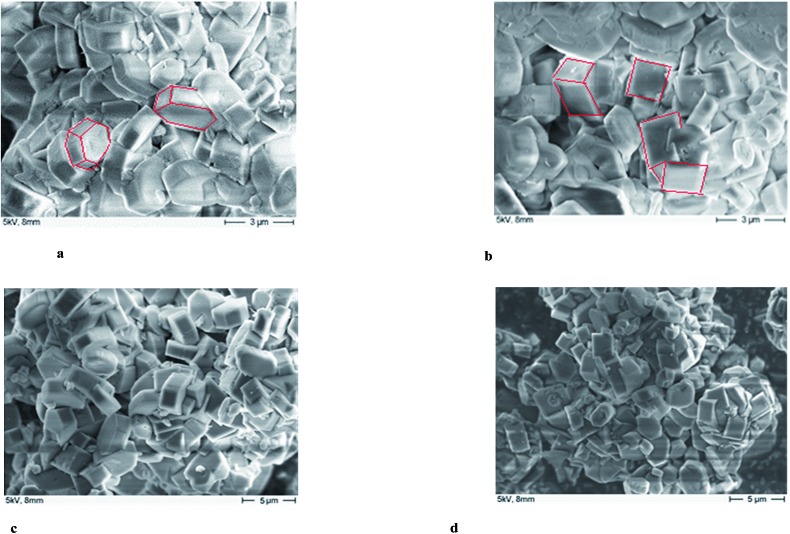
(a) SEM image of the parent H-ZSM-5. (b) SEM image of H-ZSM-5 with 7.5 wt% MoO_3_-400 °C dispersion. (c) SEM image of the parent H-ZSM-5 (post reaction). (d) SEM image of H-ZSM-5 with 7.5 wt% MoO_3_-400 °C (post reaction).

The morphology and surface properties of the parent H-ZSM-5 and MoO_3_/H-ZSM-5 (upon the best catalytic performance, the 400 °C 7.5 wt% MoO_3_ loaded sample was taken as a representative example) were compared for samples both before and after catalytic reaction using SEM and the results are presented in Fig. 7a–d. The parent H-ZSM-5 (without any Mo loadings) was pretreated under the same thermal conditions as the MoO_3_/H-ZSM-5 samples since calcination may also affect the zeolite crystal structure. Fig. 7b shows that there are no observable, individual bulk MoO_3_ particles on the surface of ZSM-5 even at 7.5 wt% loading level. As suggested by Tessonnier *et al.*,^17^ bulk MoO_3_ crystal often appears in a clearly defined platelet-like shape of several micro meters in length and can be easily identified by the SEM. The smooth surface of the zeolite crystals shows an effective dispersion of Mo species over the zeolite crystal surface. For samples prepared under these conditions, comparison between Fig. 7a and b shows that the crystal size of H-ZSM-5 did not change significantly after loading the Mo species. However, it is possibly seen that there are more cubic zeolite particles in the SEM images of the MoO_3_/H-ZSM-5 while more hexagonal crystals are present in the SEM images of the parent H-ZSM-5. The Mo species is considered to slightly modify the morphology and surface texture of the zeolite crystals, probably during the calcination process.

### Transmission electron microscopy (TEM)

**Fig. 8:**
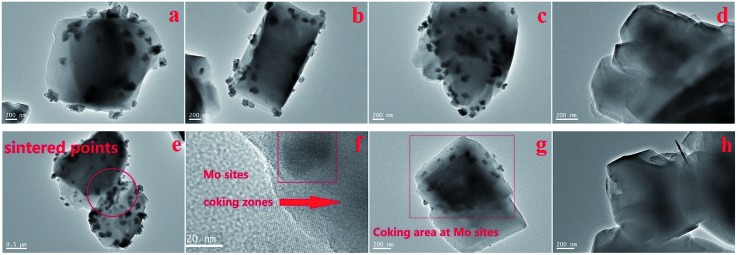
TEM images of post catalytic-testing samples with different MoO_3_ loadings, (a) 5 wt%, (b) 7.5 wt%, (c) 10 wt% and (d) parent H-ZSM-5, (e) sintering effects (5 wt%), (f) amplified coking areas near the Mo species (5 wt%), (g) coking areas near Mo species (5 wt%) and (h) parent H-ZSM-5, all the samples are prepared at 400 °C and the MTH reaction proceeded at 400 °C More images of other samples are included in the Fig. S1–S9.†

The morphology of the Mo distribution and coke deposition over the post catalytic run samples are shown in the TEM pictures (Fig. 8a–h). The outer clusters of Mo species (different with inner channel Mo species, suggested possibly as mainly MoO_3_ particles covered by coke)^21–25^ are uniformly dispersed over the zeolite surface (with no significant overlapping of particles and located on the same layer). These TEM-observable clusters are not easily detected by SEM, having diameters of 20–30 nm. Their distribution is significantly more noticeable on the 10 wt%/MoO_3_ sample (Fig. 8c), giving support to the observed peak of MoO_3_ in the XRD results (Fig. 2b). Some zeolite particles appear to be linked by the Mo clusters, as seen in Fig. 8e, possibly due to sintering effects. The large areas of diffuse black or grey color are due to the carbon deposition (coke species), which blocks the zeolite channels and finally leads to the deactivation of the catalyst. Interestingly, the coking zones are always located close to, or at, the molybdenum species clusters (Fig. 8f and g). In contrast, the coking zones on the parent H-ZSM-5 after catalytic reaction are more homogeneously dispersed (Fig. 8d and h). A possible explanation could be the Mo containing clusters give rise to the formation of (or adsorb) the formed coke species during the MTH reaction. The covering Mo species on the zeolite external surface may also block the zeolite channels, and contribute to an early deactivation of the catalyst. Due to the resolution limitation of the instrument, the inner channel Mo species are not observable. However, the migration of Mo species into the inner zeolite channel is still supported by our NH_3_-TPD and FT-IR results. It is reasonable that those surface Mo sites are where the Mo species migrate into the zeolite channels. Thus, the inner Mo based active sites should also be taken into account for the resulted coke formation. The promoting effect of Mo species inside the zeolite channel at or near Brønsted acid sites for olefin aromatization and coke formation have been reported previously.^17,19,20,22,28^

### Brunauer–Emmett–Teller (BET) measurements

The BET results of the fresh and post-reaction samples are shown in Table 1.

**Table 1:** BET analysis on fresh and post catalytic run H-ZSM-5 samples^*a*^

Sample (and loading)	Specific surface area/(m^2^ g^–1^)	Pore volume/(cm^3^ g^–1^)
H ZSM-5	323.6	0.182
5 wt% MoO_3_	309.5	0.177
7.5 wt% MoO_3_	302.9	0.174
7.5 wt% MoO_3_-500 °C	295.1	0.168
10% MoO_3_	253.0	0.149
*Post-run* ZSM-5	17.6	0.018
*Post-run* 5 wt% MoO_3_	9.0	0.014
*Post-run* 7.5 wt% MoO_3_	5.3	0.012
*Post-run* 7.5 wt% MoO_3_-500 °C	7.5	0.014
*Post-run* 10% MoO_3_	2.4	0.008

^*a*^From calibration studies, the standard deviation of the measured parameter is set as unity in the last decimal (*e.g.* 0.182 ± 0.001 and 323.6 ± 0.1).

The measured surface areas of the HZSM-5, 5 wt% MoO_3_/ZSM-5, 7.5 wt% MoO_3_/ZSM-5 and 10 wt% MoO_3_/ZSM-5 are 323.6, 309.5, 302.9 and 253.0 m^2^ g^–1^ respectively, suggesting that the Mo loading did not significantly affect the zeolite surface area and also the pore volume, up to the 7.5 wt% loading level sample. However, significant differences in both the specific surface area and pore volume emerged when the loading level of MoO_3_ increased to 10 wt%. As suggested by Borry *et al.*,^22^ the Mo content required for a monolayer distribution of MoO_3_ on ZSM-5 is about 4–5 wt%. The distribution of MoO_3_ within this range does not significantly affect the porous properties. However, when the MoO_3_ content exceeds this level (*e.g.* 10 wt% of MoO_3_), it appears that the surface MoO_3_ species might block some of the zeolite pores, leading to the reduction and partition of the internal surface area. The surface area and pore volume of 7.5 wt% MoO_3_ H-ZSM-5 (500 °C) are smaller before reaction, but correspondingly larger after reaction, as compared with the 7.5 wt% MoO_3_ H-ZSM-5 (400 °C) sample.

## Catalytic properties

### Selectivity to benzene, toluene and xylenes (BTX)

After 5 hours of operating the continuous MTH reaction at a temperature of 400 °C, the original H-ZSM-5 catalyst achieved nearly 100% methanol conversion with only trace amount of methanol left in the liquid product. Among all the MoO_3_ loaded samples (400 °C prepared), the 5 wt% MoO_3_ loaded sample exhibited the highest methanol conversion (about 87%); the catalysts of higher MoO_3_ loading levels showed lower conversion of methanol (about 80% by the 7.5 wt% MoO_3_ loaded sample, 67% by the 10 wt% MoO_3_ loaded sample) due to faster deactivation of the catalyst.

**Fig. 9:**
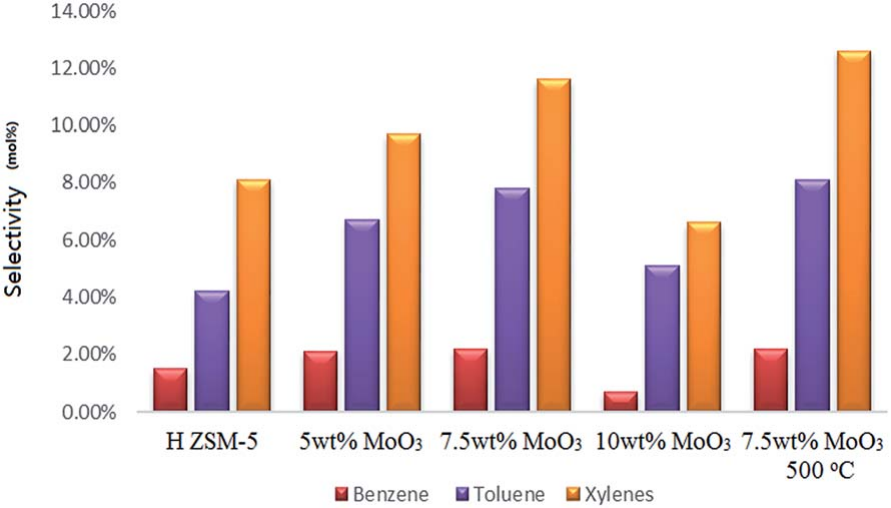
Selectivity to benzene, toluene and xylenes after a 5 h catalytic run; WHSV = 2 h^–1^, catalyst bed temperature 400 °C, and atmosphere pressure conditions.

**Fig. 10:**
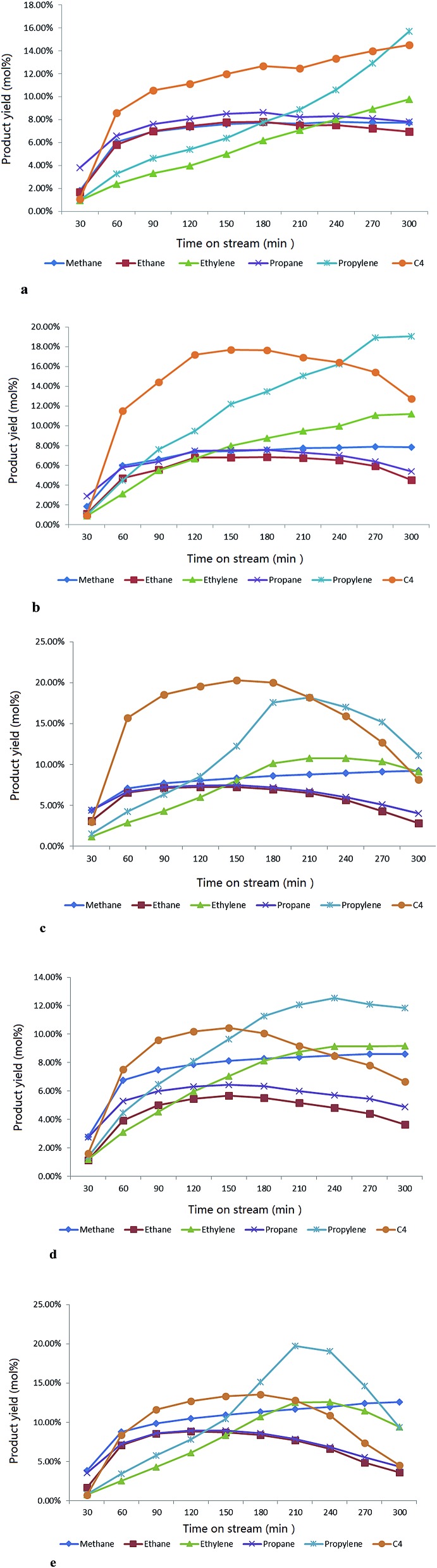
(a) The evolution of various gas products with time-on-stream over the parent H-ZSM-5 at a temperature of 400 °C, atmosphere pressure. (b) The evolution of various gas products with time-on-stream over 5 wt% MoO_3_/H-ZSM-5 at a temperature of 400 °C, atmosphere pressure. (c) The evolution of various gas products with time on-stream-over 7.5 wt% MoO_3_/H-ZSM-5 at a temperature of 400 °C, atmosphere pressure. (d) The evolution of various gas products with time-on-stream over 10 wt% MoO_3_/H-ZSM-5 at a temperature of 400 °C, atmosphere pressure. (e) The evolution of various gas products with time-on-stream over 7.5 wt% MoO_3_/H-ZSM-5 (500 °C prepared) at a temperature of 400 °C, atmosphere pressure.

Analysis of the resulting liquid fraction revealed that it contained predominantly alkylated (poly-methyl) benzenes; very tiny amount of liquid non-aromatic hydrocarbons was also detected (those fractions might also exist in the gas output in a tiny amount, and give some difficulty for a precise quantification, but not the interest of this research). The major observation is that MoO_3_ loading on zeolites promoted a higher selectivity to the formation of benzene, toluene and xylenes (Fig. 9), although it also resulted in earlier catalyst deactivation probably by the enhanced product accumulation. The highest BTX selectivity was achieved from the 7.5 wt% MoO_3_ loading sample even though its catalytic activity was partially lost by the end of the reaction time (as is reflected by the reduced gas yields shown in Fig. 10c). Interestingly, an increase in MoO_3_ dosage, doping from 7.5 wt% to 10 wt% resulted in a noticeable drop in the selectivity to the targeted BTX aromatic products. This is possibly due to the loaded Mo species possessing a more obvious pore-blocking effect at the highest doping level (10 wt%). It is clear that the reduced available channel-opening areas by Mo species over the zeolite surface (as is reflected by the BET results), contributed to the higher selectivity to some BTX products at a lower doping level of MoO_3_ but, finally, limited the product transportation at the higher loading levels. The 7.5% wt MoO_3_ sample calcined at 500 °C has slightly higher selectivity to toluene and total xylenes, but its methanol conversion rate (about 69%) is much lower.

### Gaseous olefin time-on-stream (TOS) yield

The major gaseous hydrocarbon production in time-on-stream for each sample is shown in the Fig. 10a–e.

The parent H-ZSM-5 exhibited gradually increasing gas olefin yield through the whole reaction time (the separation of butanes and butylenes was limited by our instrument; herein we report the total C_4_ yield). In contrast, the samples with 5 wt% and 7.5 wt% MoO_3_ achieved apparently higher propylene and C_4_ hydrocarbon yields (the enhanced C_4_ olefin yield is supposed to contribute to the total C_4_ production) in the first 150 min period, especially for propylene (about 6 mol% over H-ZSM-5 *vs.* about 12.3 mol% over 7.5 wt% MoO_3_ loaded sample at the time of 150 min). Both the 7.5 wt% and 10 wt% MoO_3_ loaded catalysts experienced a peak period of the total gas production at or near 210 min, and subsequently became less active. The performance of these two samples implies an early deactivation of the catalyst, possibly by accelerated coke formation (Fig. 8). The over-loading of MoO_3_ (10 wt%) clearly hindered the catalyst performance, as reflected by the inhibited gas yields (Fig. 10d). It should be noted that before approaching the partially deactivated status at the end of the reaction time, the 7.5 wt% MoO_3_/H-ZSM-5 sample showed noticeably higher propylene and total C_4_ yields in time on stream than all other samples tested. On the other hand, the ethylene time-on-stream yield also was improved by a moderate MoO_3_ loading but with relatively less enhancements. Both the 5–7.5 wt% MoO_3_ H-ZSM-5 catalysts reached an ethylene instantaneous yield of about 10 mol% at the end of the reaction.

Another parallel test on the 7.5 wt% H-ZSM-5 prepared *via* 500 °C calcination was demonstrated in the Fig. 10e, the reaction conditions were the same as other samples (400 °C). More obvious catalyst deactivation was observed starting at around 210 min, before which an increase on the olefin yields especially for propylene was also monitored in the time period of 30–180 min.

### Thermogravimetric analysis (TGA)

**Fig. 11:**
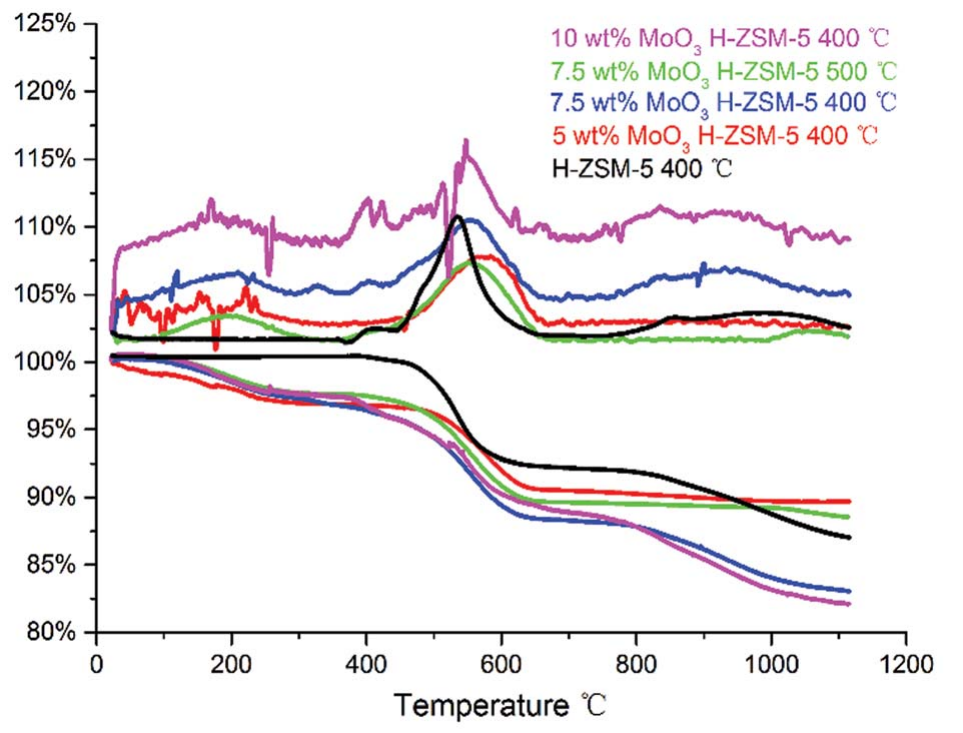
TG–DTA plot of the spent catalysts, in the temperature range of 20–1120 °C, included DTA (the top) and TG (the bottom) profiles.

TGA of the spent catalysts was carried out in air (100 ml min^–1^), in the temperature range of 20–1100 °C to study coke deposition on the spent catalysts (Fig. 11).

As illustrated in Fig. 11, the weight loss stage of 400–600 °C in the TGA profile mainly represents the onset of ‘hard coke’ formation on the spent catalyst, which has been considered to be the major reason for catalyst deactivation.^34^ Accordingly, the derivative thermogravimetry (DTG) curves also show a significant peak in this temperature range. The results shows that the 10 wt% MoO_3_ H-ZSM-5 sample contains more coke than other catalyst samples (reflected by the total weight loss of about 17.5 wt%), and the 7.5 wt% MoO_3_ H-ZSM-5 sample (400 °C) shows slightly less coke deposits. Interestingly, the 7.5 wt% MoO_3_ H-ZSM-5 prepared at 500 °C has apparently smaller amounts of coke formation. A possible explanation would be the higher temperature employed in the catalyst calcination resulted in more loss of the zeolite acid sites, on which the coke formation originally occurs. This is also supported by our FT-IR results of the as-prepared catalysts, which shows less amount of Brønsted acid sites on the 500 °C prepared 7.5 wt% H-ZSM-5. The enhanced coke formation on the 10 wt% H-ZSM-5 (400 °C) sample is more complex. Although the reduced number of inner Brønsted acid sites also slows down the coke formation from the outset of the reaction, the corresponding blocking effects on the diffusion of large molecule products (*e.g.* aromatics) by the over-loaded surface Mo species appears also to play an important role. In this case, the products are more easily trapped within the zeolite channels and finally converted into coke.

## Discussions

Recent investigations^7,16,26,35^ on C_2_/C_3_ olefin selectivity of the MTH process using H-ZSM-5 proposed a dual-pathway scheme for the olefin generation, centered on (1) a so-called “classic route” in which ethylene and propylene are eliminated from the hydrocarbon pool molecules by de-alkylation, and (2) formation of C_3_^+^ olefins also *via* intra conversion (*e.g.* methylation & cracking) of the previously formed olefin products. Svelle^16^ pointed out that the second route played an important role for the yield of propylene and other higher olefins.

The above findings can help to explain the observed promoted catalysis achieved by the MoO_3_ loaded samples (Fig. 9 and 10). The earlier enhanced propylene and C_4_ hydrocarbon yield (here we make the assumption that the enhanced butylene yield might have contributed to the total C_4_) can be attributed to MoO_3_ species effectively transported into the zeolite channels. There, the inner Mo species initiate the activation of C–H bonds for chain growth methylation as well as the cracking of larger molecules,^20,36,37^ resulting in enhanced propylene and butylene (part of the total C_4_ hydrocarbons) production in the MTH process. The activation of the C–H bond and the resulting hydrocarbon transformation promoted by Mo based catalysts have been investigated previously.^38–41^ A possible explanation is the reduction of the MoO_3_ precursor by both H_2_ and alkanes, resulting in molybdenum carbide formation which promotes the dissociation of the C–H connection. One study on CH_4_ dehydro-aromatization with co-fed H_2_ and methanol employed higher temperatures (600 °C for calcination, and up to 500 °C for reaction), and reported the formation of molybdenum carbide as well as its attendant well-recognized catalytic behavior in promoting the formation of aromatics and methylation on benzene-ring structure.^42^ However, it was reported that a higher temperature, some 800 °C or above, is required for efficient carburization of MoO_*x*_ to the corresponding carbide species.^43^ Direct conversion of MoO_3_ into molybdenum carbides at 400 °C (673 K) was in fact demonstrated by other researchers,^44^ although high pressure (some 379 bar) was required for carbide formation.

The highly-surface-dispersed nature and inner migration of the active Mo species particularly make it difficult to detect a short time period ‘transition phase’ of the Mo species during the catalytic process. Of course, our results are for completed catalytic reactions and there is a clear need for a future *in situ* investigation. An earlier report has highlighted molybdenum oxycarbide (MoOC_*x*_) or oxide carbide as a ‘transition state’ formed from the molybdenum oxide by partial reduction with methane and ethylene in a close temperature range.^45^ During methanol conversion, CH_4_ and H_2_, as well as other low carbon number alkanes, are continuously generated as typical products (they are also potential reductants), simultaneously with olefins; thus, we propose that molybdenum oxycarbide as a transition-state resultant of the MoO_3_/MoO_*x*_ precursors is formed inside the zeolite channels by partial reduction of molybdenum oxide species with the already-formed olefins, alkanes and H_2_. This catalytic material has previously been demonstrated to polarize the C–H bond leading to further bond dissociation.^22^ In a comprehensive study of Fischer–Tropsch catalytic processes, Adesina and co-workers reported a two-stage transformation of Mo oxide to MoC_1–*x*_ carbide *via* an oxycarbide phase formed in the temperature range of 380–480 °C from a MoO_3_ precursor (by reduction with alkanes and hydrogen), in agreement with our results.^46^

The promoting effects of the inner channel Mo sites on aromatization also contributed to the formation of aromatic products, as previously reported elsewhere.^16–22^ We propose also that the Mo species dispersed on the zeolite external surface also plays an important role in blocking the diffusion (out from the zeolite channels) of aromatic molecules at, or near, the zeolite channel openings (The blocking effects are simply demonstrated in the Fig. S10 and S11†). While the inner channel Mo species promotes an early-time accelerated catalytic reaction in the transition-state form of molybdenum oxycarbide, the MoO_3_ clusters/oligomers located near/at the zeolite channel openings also reduce the access to the inner channel space. In this case, reactant methanol can still move into the zeolite channels; but products of larger molecules, such as aromatics, will have a more difficult diffusion process due to this ‘physical’ blocking process. Furthermore, any aromatic ring structures with an ‘electron rich’ property may be attractive to the Mo^6+^ cations; therefore, the aromatic products could also be chemically trapped by the MoO_3_ species (or other kind of Mo^6+^ structures involved in the reaction) at the zeolite channel openings. It is particularly noteworthy that the blocking of zeolite channels by both reaction product accumulation and anchored molybdenum oxide, and other species has a considerable influence on the liquid product distribution, *e.g.* larger products such as tri-methyl benzene with more alkyl groups are more likely to be trapped inside the zeolite channels, leading to our observed higher selectivity to benzene, toluene and xylenes (Fig. 9).

Any resulting product transportation out of the inner space will become increasingly more difficult with time-on-stream. More olefins trapped inside the zeolite channels would go through the oligomerization and dehydrogenation steps (aromatization), finally converting into aromatics. The previously enhanced olefin yield (Fig. 10c and d) would naturally fall with attendant higher selectivity to aromatics, further accelerating the accumulation of aromatic products at the zeolite channel openings. These trapped aromatics finally become the precursors of coke species. This is in agreement with the hypothesis of Mores' *et al.* that coke is formed at specific positions near the zeolite surface.^47^ The incorporation of Mo active sites in the catalytic system further speeds up the above coking process, while promoting the formation of valuable products. On the other hand, the resulted loss of zeolite Brønsted acidity by Mo species will also affect the catalyst lifetime in the MTH process.

The unique catalytic performance (observed highest selectivity to BTX, and early enhanced yield of valuable propylene and C_4_ products) of the 400 °C prepared 7.5 wt% MoO_3_/H-ZSM-5 is mainly ascribed to a proper balance between the number of resulted inner channel Mo catalytic sites and the maintained intrinsic zeolite Brønsted acidity observed in our series of characterizations. It is clear that the higher temperature (500 °C) synthesis induced a faster inner migration of the Mo species, leading to more obvious loss of zeolite Brønsted acid sites, which may be more difficult to control at this temperature. We propose that a moderated lower calcination temperature (400 °C) employed in catalyst preparation gives more freedom to approach such a balanced status, and optimization of the prepared catalyst with metal oxide loadings.

We finally return to Fig. 7c and d where we have shown SEM images of the post-reaction samples of H-ZSM-5 and 400 °C prepared 7.5 wt% MoO_3_/H-ZSM-5 (the latter has best performance as we mentioned above). It is apparent that the particles of post-reaction MoO_3_/H-ZSM-5 are considerably more compacted, while the particles of the post-reaction H-ZSM-5 are comparatively ‘looser’. We attribute this difference in crystal morphologies to the sintering effect (also shown by TEM, Fig. 8e) of the Mo species on the external zeolite surface. The correspondingly reduced intra-crystalline space also accelerated the catalyst deactivation.

The surface physical characteristics of the post-run catalysts are also shown in Table 1 (BET). It can be seen that on these samples, both surface area and pore volume were reduced dramatically, most certainly due to coke formation.

## Conclusion

A series of MoO_3_/H-ZSM-5 catalysts have been prepared at a temperature of 400 °C, lower than the conventional preparation temperature, in an attempt to optimize the dispersion of MoO_3_/MoO_*x*_ both on, and also within, the zeolite host. The MoO_3_ structure was successfully formed on the surface of prepared samples, as reflected by the XRD and Raman results. The SEM and TEM results support a well uniform dispersion of the Mo species on both fresh samples and coked samples. The FT-IR and NH_3_-TPD analysis showed that a small portion of MoO_3_ species also migrated into the zeolite channels with increasing loading levels of MoO_3_. In addition, samples prepared at the (conventional) higher temperature (500 °C) leads to enhanced MoO_3_ migration into the zeolite channel, with further loss of Brønsted acidity. The calcination temperature and MoO_3_ concentration both exert important influences on the distribution of Mo species, and their resulting activities in the zeolite catalyst system.

To achieve a satisfactory catalysis performance, a MoO_3_ loading level no higher than 7.5 wt% was required. An enhanced propylene and C_4_ gas product generation in the early period of the reaction was observed on the MoO_3_ modified samples and subsequently converted to the improved aromatic production at the later stages of reaction. C–H bond activation by Mo species (a transition period catalytic material we believe to be Mo–Oxycarbide) inside the zeolite channels is proposed as the mechanism for the promoted catalysis *via* the olefins methylation and intra-conversion process; importantly, this situation benefits both the gas olefin yield and also the final BTX selectivity. The Mo species on the zeolite external surface also affected the final product distribution by modifying the zeolite channel openings. The enhanced product accumulation inside the zeolite channel finally resulted in a premature deactivating behavior of the catalyst system.

This work illustrates the high degree of control possible in these catalytic processes by a careful combination of synthesis temperatures and MoO_3_ loading levels, where a balance of targeted catalytic properties can be optimized.
