# Supplementary material for: Methanol-to-hydrocarbons conversion over MoO3/H-ZSM-5 catalysts prepared via lower temperature calcination: a route to tailor the distribution and evolution of promoter Mo species, and their corresponding catalytic properties
Source: Chem Sci. 2015 Jun 11;6(9):5152–63. doi: 10.1039/c5sc01825k (PMC5667184; doi:10.1039/c5sc01825k)
Supplement: Supplementary file 2 [file SC-006-C5SC01825K-s002.pdf]

Electronic Supplementary Material (ESI) for Chemical Science.
This journal is © The Royal Society of Chemistry 2015

Methanol-to-hydrocarbons conversion over MoO_3/H-ZSM-5 catalysts prepared via lower temperature calcination: A route to tailor the distribution and evolution of promoter Mo species, and their corresponding catalytic properties

Bonan Liu[a], Liam France[a], Chen Wu[c], Zheng Jiang[a], Vladimir L Kuznetsov[a], Hamid AlMegren[b], Mohammed AlKinany[b], Saud A Aldrees[b], Tiancun Xiao[a*], Peter P Edwards[a**]

The supplementary data have included a full range of TEM pictures of the post-run samples, including 400 ^{o}C calcinated MoO_3/H-ZSM-5 samples (coked) and the parent H-ZSM-5 (coked), showing the distribution of Mo clusters on the zeolite crystallite external surface, and the observed coke deposition upon the spreading of the Mo species. The Figures S1-S9 are the complementary of the Fig. 8 in the paper. TEM measurements were undertaken using a JEM-2100UHR microscope (200kV).

The pure H-ZSM-5 sample after MTH reaction exhibits more uniformly dispersed coking zones (or areas), as shown by the Fig. S1 and S2. Clusters of Mo species on the zeolite external surface are clearly shown for the post-run MoO_3 loaded samples. Those black-color, uniformly surface dispersed particles (better shown in the Fig. S3, S5, and S7) have covered some areas of the zeolite crystal surface, and are supposed to partially block the product transportations. On the other hand, the coking zones of the MoO_3 loaded samples also show somewhat preference to the location of those surface Mo clusters (better shown in the Fig. S9). Amplified vision (Fig. S4, S6 and S8) also supports the above assumptions.

Based on the TEM observations, a simple visual 'reoccurrence' of the suspected blocking effects, by the external surface Mo species in the catalytic reactions is drawn with Chemical Bio3D Ultra (Perkin Elmer, 14.0.0.117). In the figures S10 and S11, a standard 'MFI.cif' is employed for demonstrating the zeolite framework, representing the regular zeolite channel opening structures

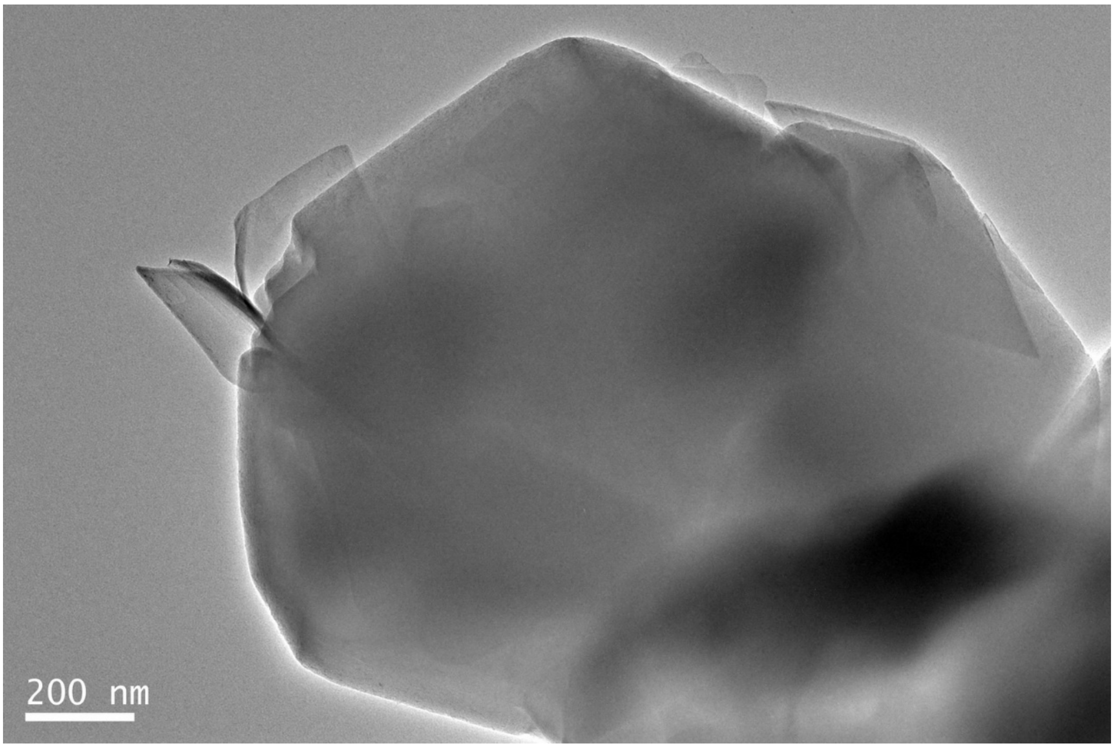

Figure. S1 TEM images of post catalytic-testing parent H-ZSM-5

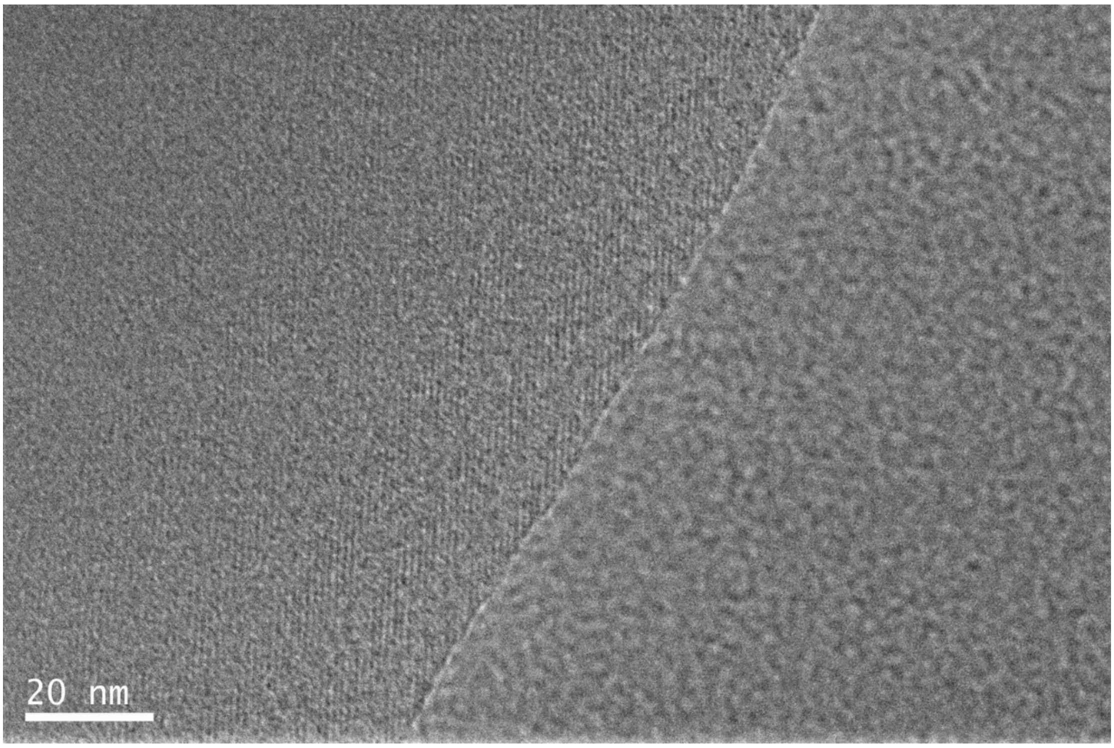

Figure. S2 TEM images (Amplified) of post catalytic-testing parent H-ZSM-5

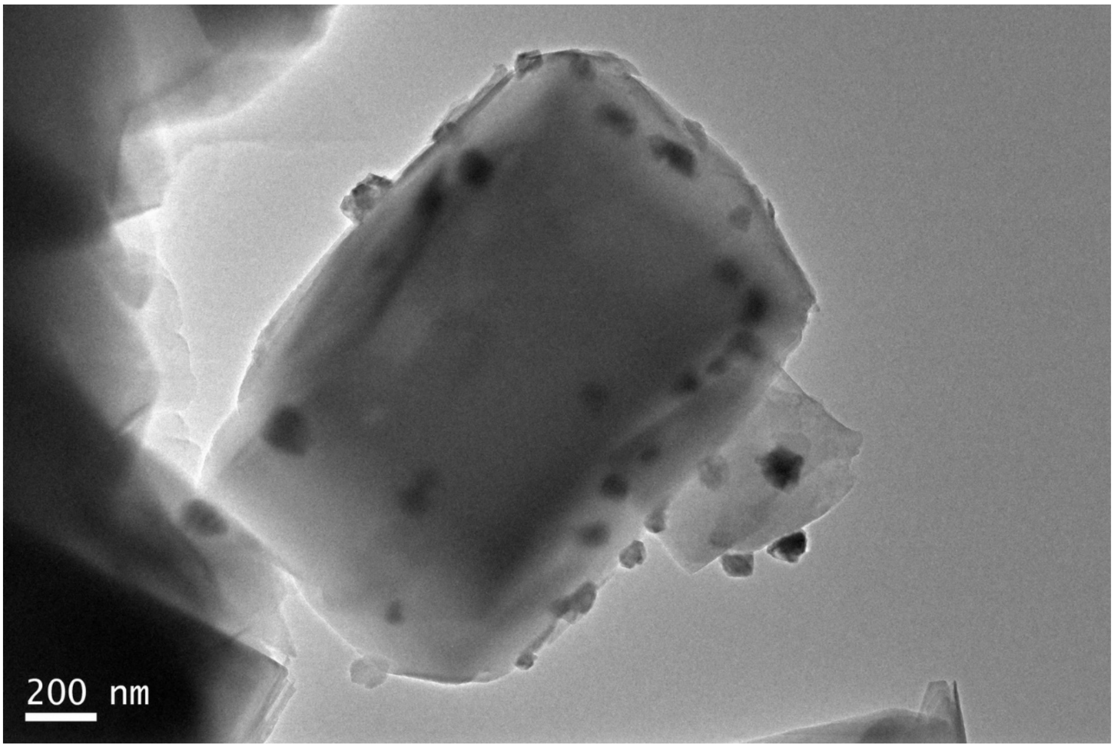

Figure. S3 TEM images of post catalytic-testing 5wt% MoO_3 H-ZSM-5

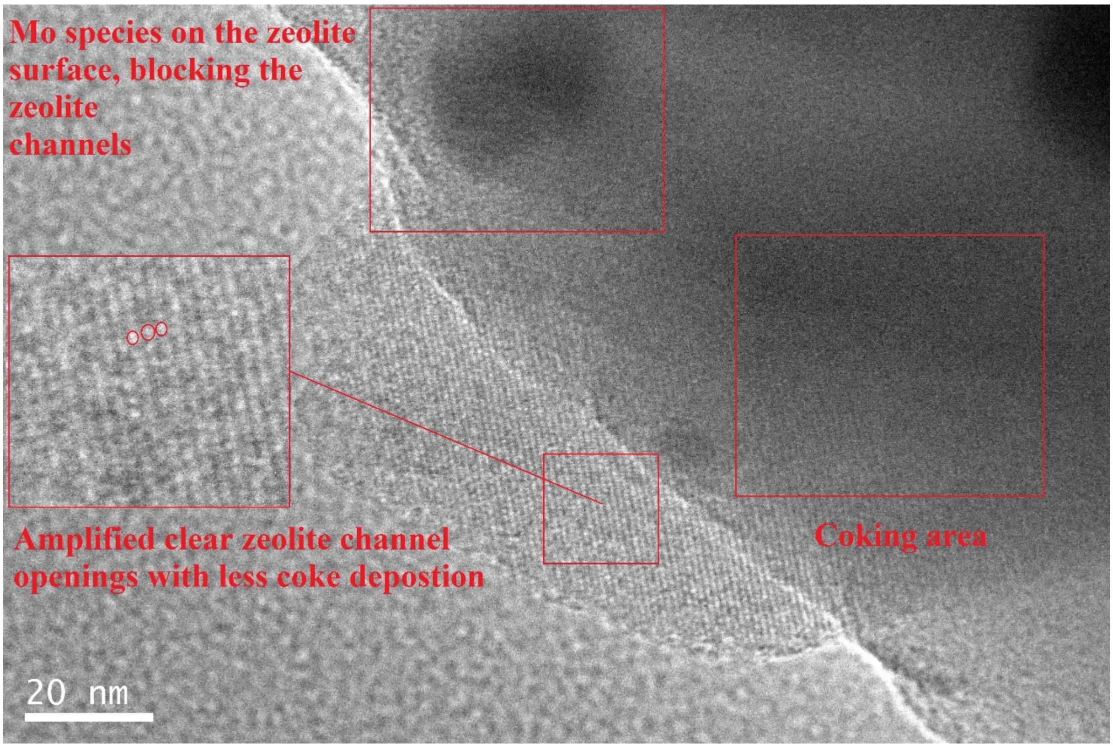

Figure. S4 TEM images (Amplified) of post catalytic-testing 5wt% MoO_3 H-ZSM-5

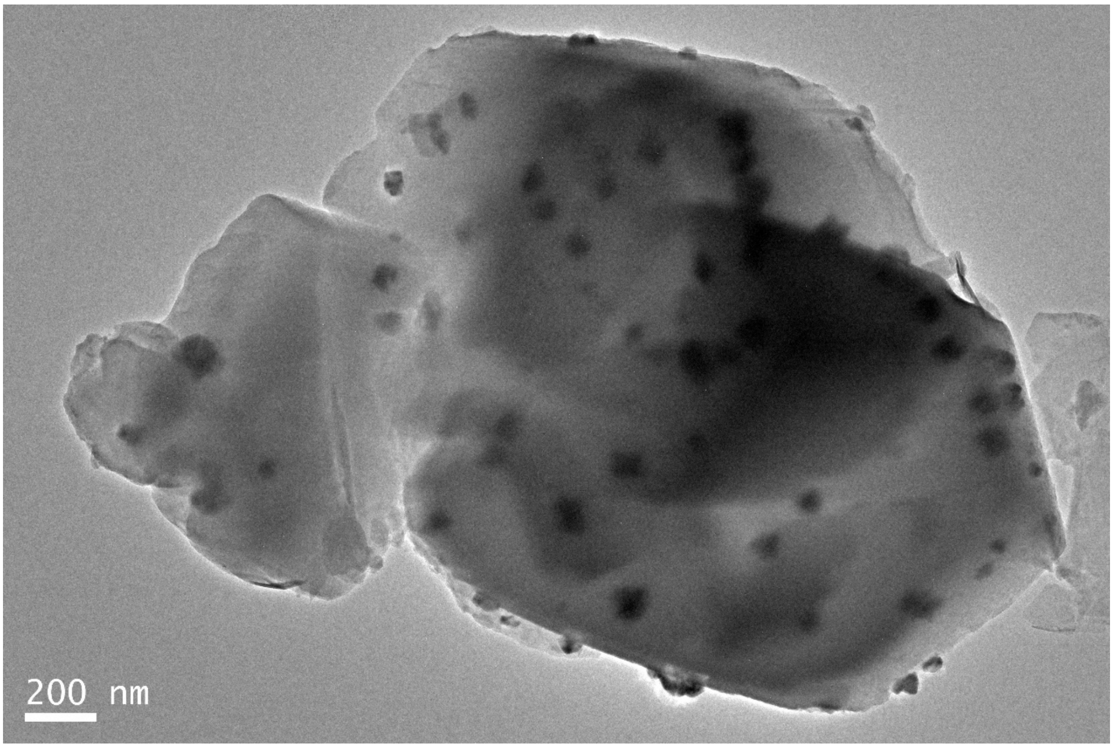

Figure. S5 TEM images of post catalytic-testing 7.5wt% MoO_3 H-ZSM-5

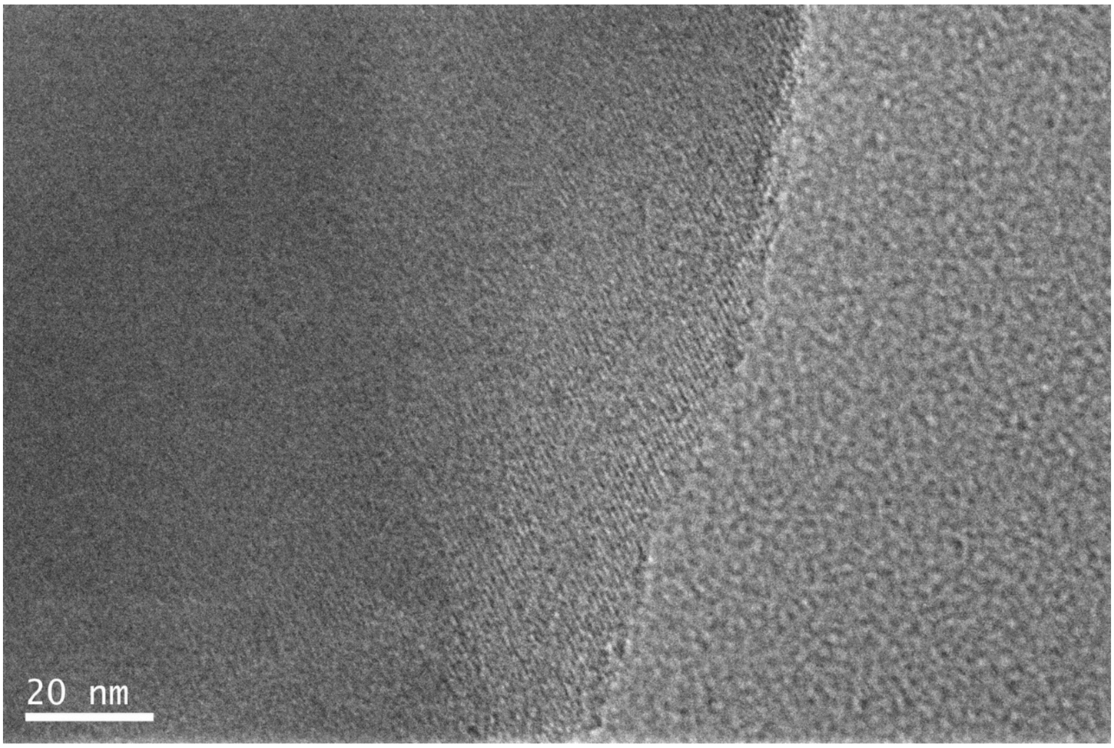

Figure. S6 TEM images (Amplified) of post catalytic-testing 7.5wt% MoO_3 H-ZSM-5

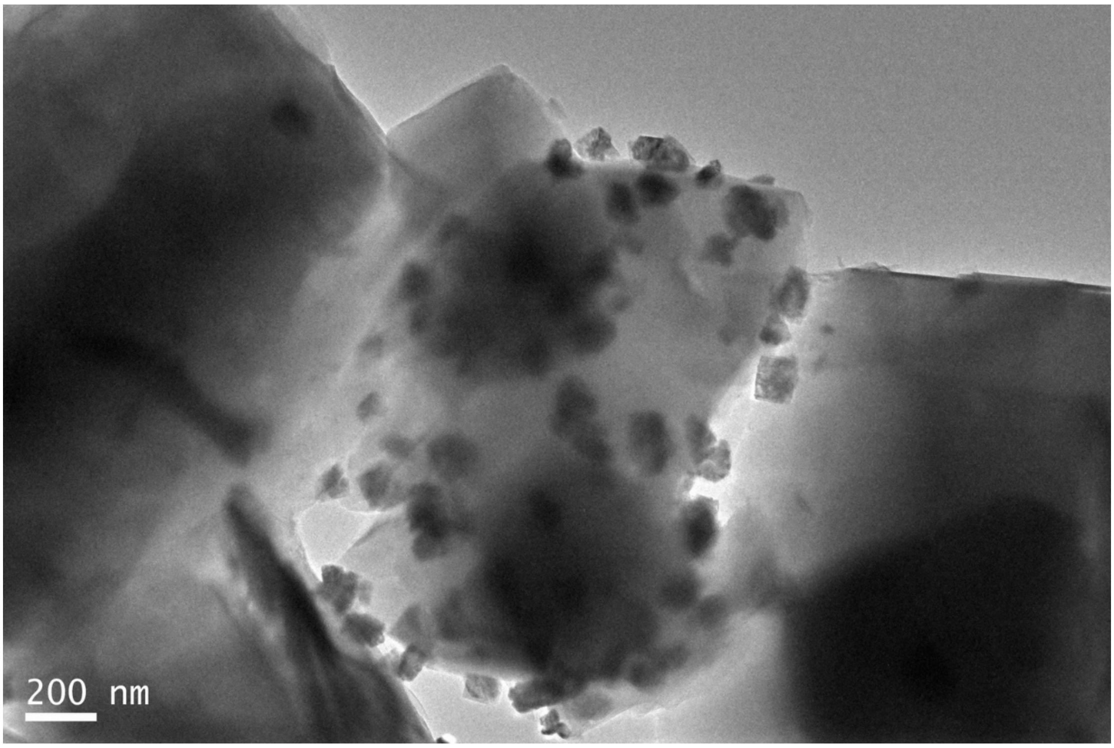

Figure. S7 TEM images of post catalytic-testing 10wt% MoO_3 H-ZSM-5

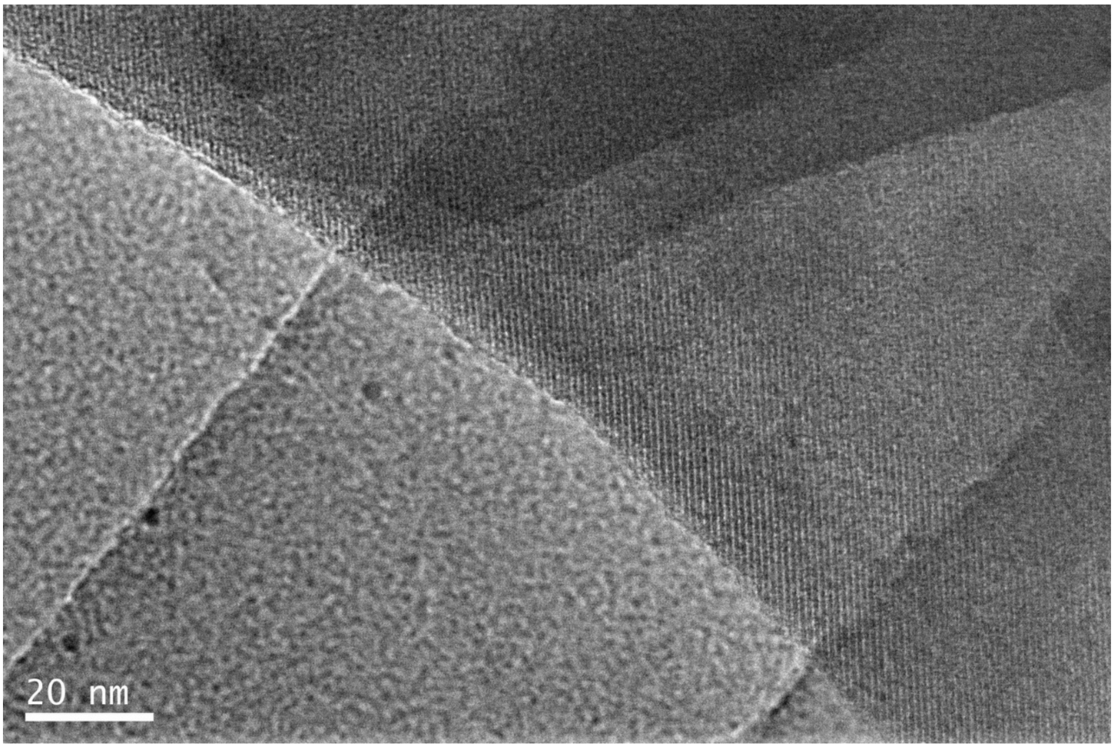

Figure. S8 TEM images (Amplified) of post catalytic-testing 10wt% MoO_3 H-ZSM-5

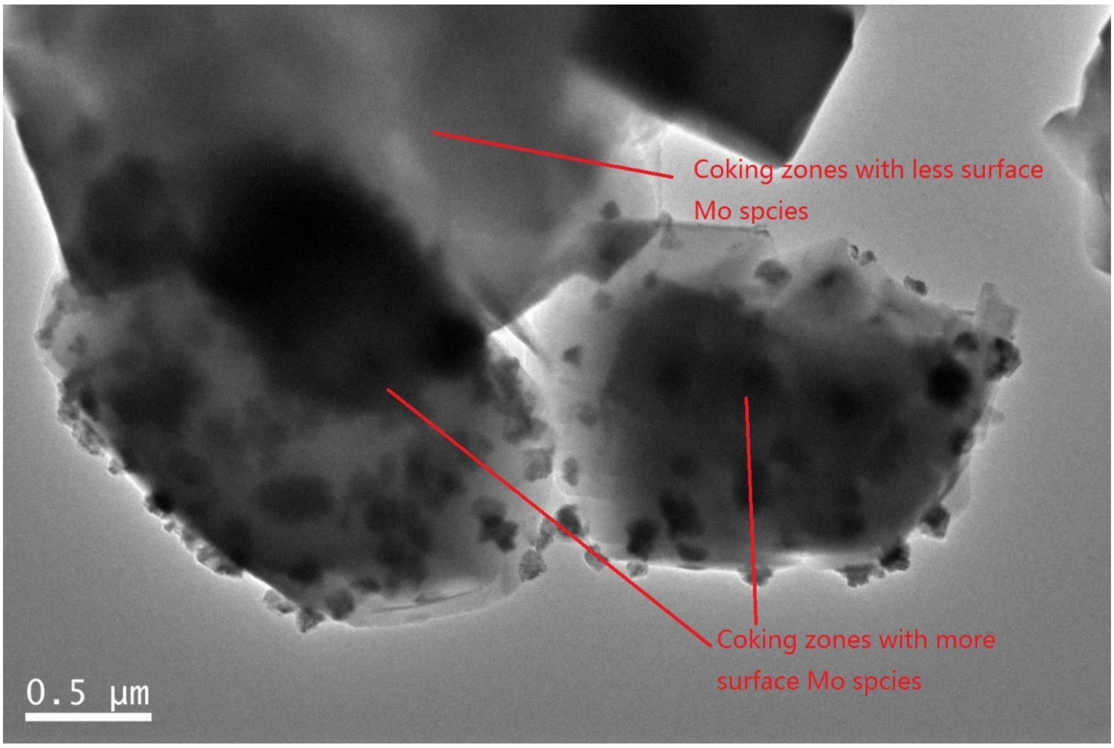

Figure. S9 TEM images of post catalytic-testing 7.5wt% MoO_3 H-ZSM-5

The selected TEM image happens to have captured 3 zeolite crystal particles. The bottom two particles possess more Mo species clusters on the surface; thus, have obtained more black coking zones.

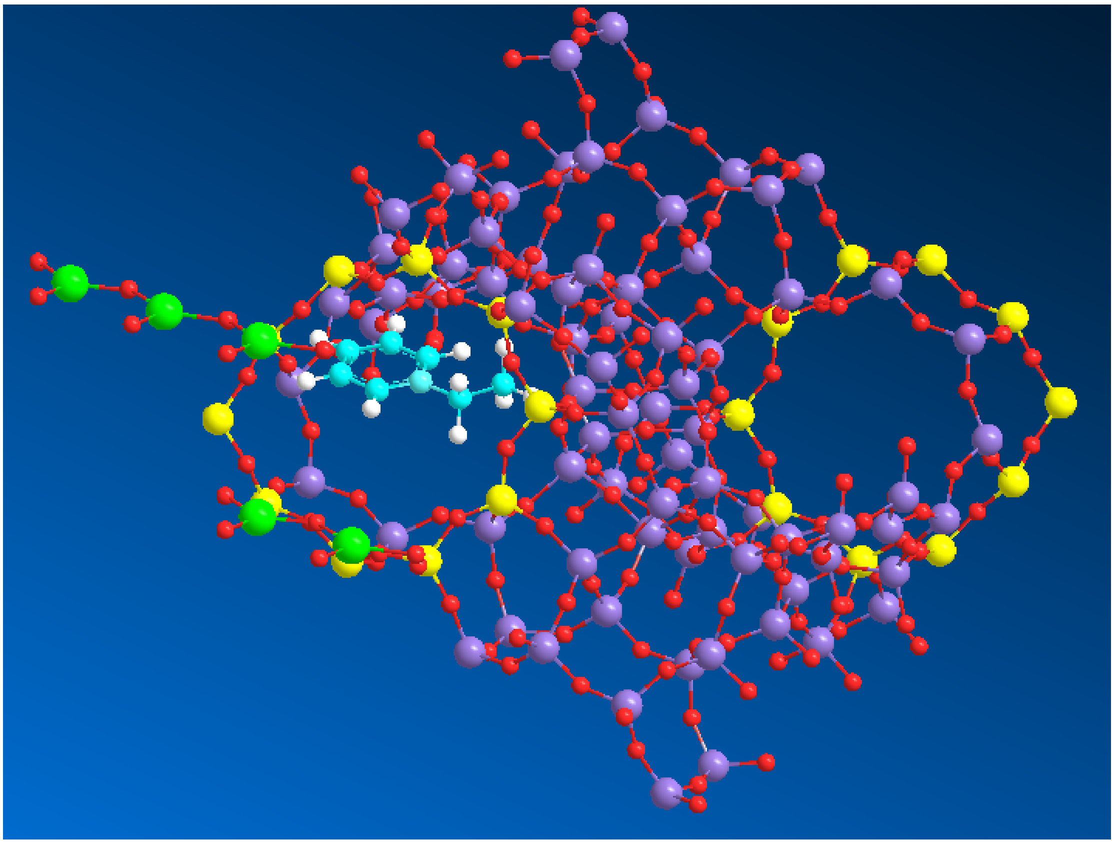

Figure. S10 Demonstration of blocking effects by surface Mo species (MoO_3 clusters as suspected) of the 7.5wt% MoO_3 H-ZSM-5 on the transportation of a toluene molecule.

The zeolite framework cations are drawn in violet color, and oxygen atoms are in red color. The 10-member ring of the MFI channel openings is highlighted with yellow color. A generated toluene molecule shown in light blue color is being transported from the inner channel to the outside, and directly facing the surface Mo clusters. The suspected MoO_3 clusters (the number of MoO_3/MoOx units could be more than the demonstrated 3 in reality) located on the external zeolite surface at near channel opening positions are highlighted (Mo atoms are shown in bright green color)

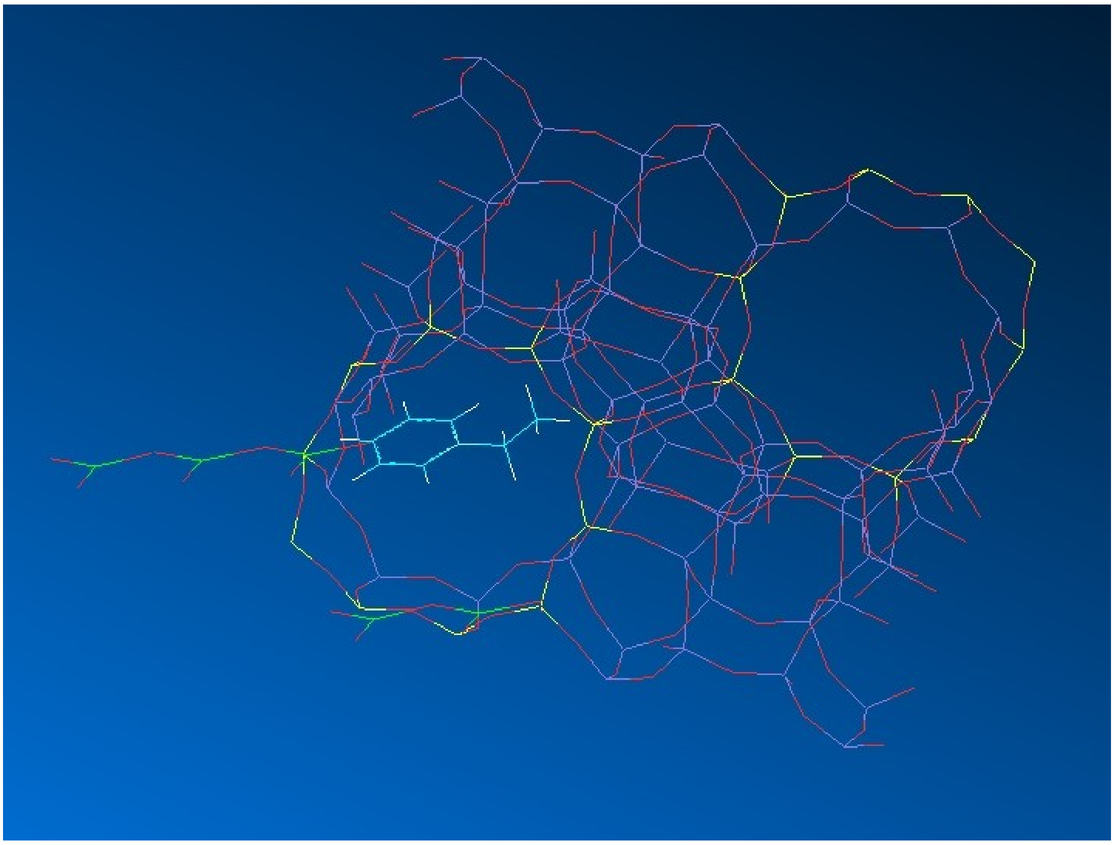

Figure. S11 Demonstration of blocking effects by surface Mo species (MoO_3 clusters as suspected) of the 7.5wt% MoO_3 H-ZSM-5 on the transportation of a toluene molecule.

The figure is simplified into a sticks display model for a clear vision.
